# Suzetrigine: A Novel Non-Opioid Analgesic for Acute Pain Management—A Review

**DOI:** 10.3390/ddc4030032

**Published:** 2025-07-04

**Authors:** Meaghan Jones, Aryanna Demery, Rami A. Al-Horani

**Affiliations:** Division of Basic Pharmaceutical Sciences, College of Pharmacy, Xavier University of Louisiana, New Orleans, LA 70125, USA

**Keywords:** acute pain, Na_v_1.8 inhibitor, non-opioid, suzetrigine

## Abstract

Suzetrigine represents a groundbreaking advancement in acute pain management as the first FDA-approved selective Na_v_1.8 inhibitor. This comprehensive review synthesizes data from clinical trials, pharmacological studies, and prescribing information to evaluate its mechanism, efficacy, safety, and clinical implications. With demonstrated superiority over placebo in pivotal trials (SPID48: 29.3–48.4; *p* < 0.0001) and a favorable safety profile devoid of opioid-like addiction risks, suzetrigine offers a much-needed alternative in the opioid crisis era. However, its modest effect size compared to full-dose opioids, CYP3A-mediated drug interactions, and limited long-term data warrant judicious use. This article provides a balanced perspective on suzetrigine’s role in modern pain management protocols.

## 1. Introduction

The opioid epidemic continues to devastate communities, particularly in the United States, as reported by the Centers for Disease Control and Prevention (CDC). In 2022, drug overdose deaths reached a record high of nearly 108,000, with approximately 82,000 involving opioids. However, signs of stabilization emerged, with declines in heroin- and prescription opioid-related deaths by 36% and 12%, respectively. In 2023, the trend began to reverse, with overall overdose deaths decreasing slightly and opioid-related deaths falling from 84,181 in 2022 to 81,083; although fentanyl deaths declined, stimulant-related deaths rose, and geographic differences were stark across states. Most significantly, provisional 2024 data suggest a sharp 27% drop in total drug overdose deaths—the lowest since 2019—with estimated opioid deaths decreasing to 54,743, a decline potentially driven by broader naloxone access, improved addiction treatment, and evolving drug use patterns [1,2]. Regardless, this public health crisis has created an urgent need for effective non-opioid analgesics, yet therapeutic innovation has lagged significantly. Before suzetrigine’s approval in 2025, the most recent FDA-approved novel pain medication was celecoxib in 1998 [3]. Suzetrigine (VX-548) represents a notable pharmacological development as the first selective Nav1.8 sodium channel blocker specifically developed for acute pain management [4]. This review synthesizes current evidence on suzetrigine’s mechanism of action, clinical efficacy, safety profile, and therapeutic potential.

## 2. Current Non-Opioid Practices for Acute Pain

Current guidelines recommend a stepwise, multimodal approach to pain management, tailored to pain severity and individual patient factors [5–7]. This strategy emphasizes combining nonpharmacologic therapies with pharmacologic options—including non-opioid medications, local/regional anesthesia, and opioids when necessary—to enhance analgesia while minimizing opioid reliance and associated harms. Acetaminophen is widely recommended for most patients, given its safety profile and versatility across oral, intravenous, and rectal routes, though hepatotoxicity limits dosing to a 4 g/day maximum. NSAIDs, both COX-2-selective (e.g., celecoxib) and non-selective (e.g., ibuprofen, ketorolac), are effective for acute pain but carry cardiovascular, renal, and gastrointestinal risks. Gabapentinoids may help patients under 75 years undergoing major surgery, but concerns over sedation and respiratory depression have tempered enthusiasm. Intravenous lidocaine and ketamine offer alternatives for surgical pain, particularly in opioid-tolerant or high-risk patients, but both require careful monitoring due to toxicity and neuropsychiatric side effects. Agents like dexamethasone and alpha-2 agonists are valuable adjuncts, especially for their antiemetic and sedative-sparing properties. Despite these options, each class has notable limitations, underscoring the need for novel, more targeted analgesics. For example, tanezumab (anti-nerve growth factor monoclonal antibody) is an emerging non-opioid treatment, particularly for bone pain associated with osteoarthritis or cancer [8]. More importantly, suzetrigine, a recently approved Nav1.8 inhibitor, represents a promising advance by selectively targeting peripheral pain pathways while potentially avoiding the systemic side effects associated with existing treatments. However, further studies are needed to confirm its long-term efficacy and safety across broader patient populations [5–7].

## 3. The Chemistry of Suzetrigine

**Figure 1.**
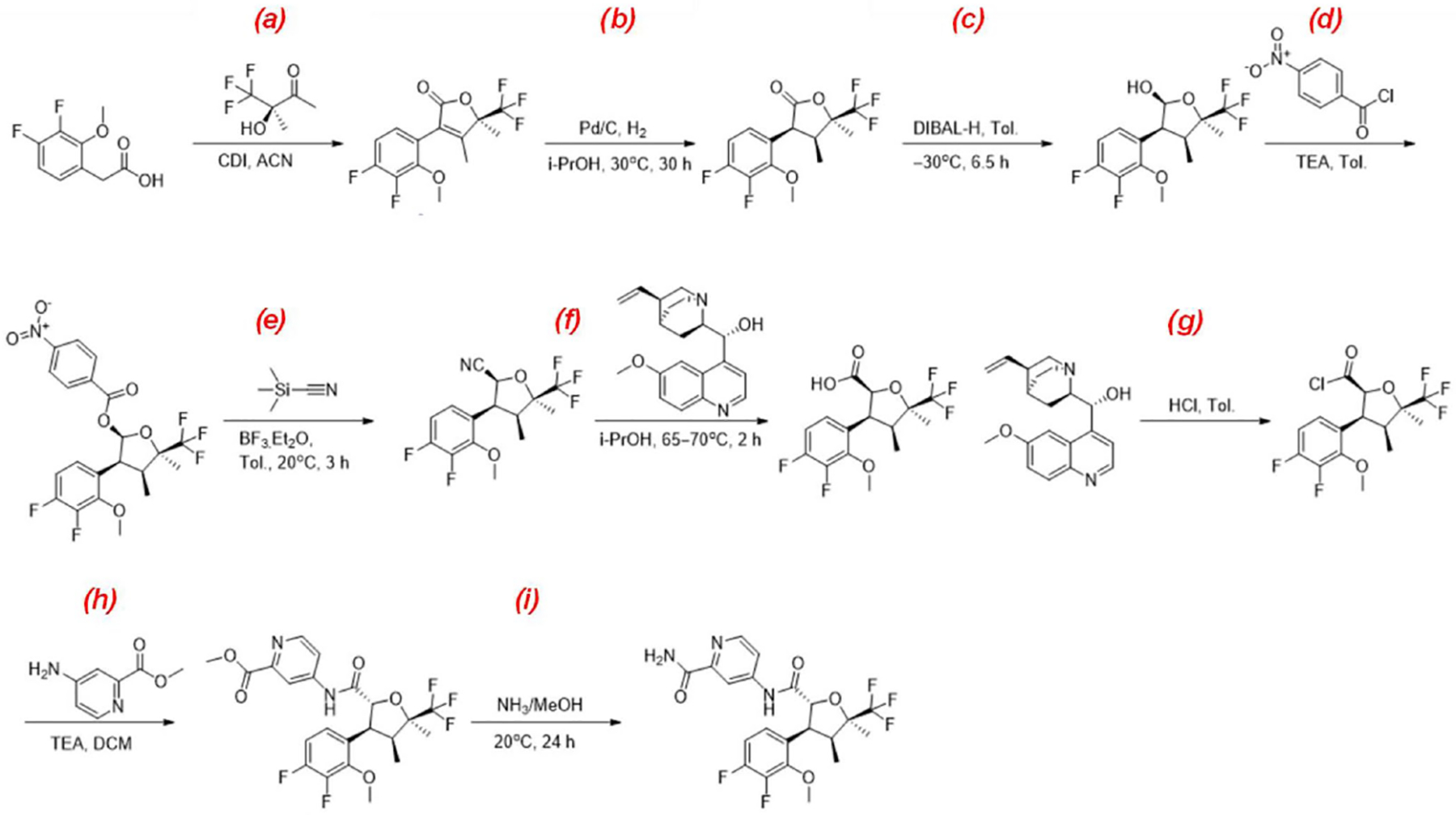
Chemical reactions: (**a**) 1,1′-carbonyldiimidazole/acetonitrile for 1.5 h at −2–0°C, then for 5 h at 35 °C; (**b**) palladium-carbon; hydrogen/isopropyl alcohol for 30 h at 30–31 °C (2585.81–11636.1 Torr); (**c**) diisobutylaluminium hydride/toluene for 6.5 h at −31–−26 °C; (**d**) triethylamine/toluene at 0 °C; (**e**) boron trifluoride diethyl etherate/toluene for 3 h at 20 °C; (**f**) isopropyl alcohol; n-heptane for 2 h at 65–70 °C; (**g**) hydrogen chloride/toluene; water at 20 °C, then for 4 h at 30 °C; (**h**) triethylamine/dichloromethane for 4 h at 25 °C; (**i**) ammonia/methanol for 24 h at 20 °C.

The drug is chemically known as 4-[(2R,3S,4S,5R)-3-(3,4-difluoro-2-methoxyphenyl)-4,5-dimethyl-5-(trifluoromethyl)oxolane-2-amido]pyridine2-carboxamide with a molecular formula of C_21_H_20_F_5_N_3_O_4_ and a molecular weight of 473.39 g/mol. Suzetrigine is a white to off-white solid and is practically insoluble in water. The chemical synthesis of the drug was reported by Vertex Pharmaceuticals in a series of patents. A summarized schematic representation is given in Figure 1 [9]. Another method was reported recently as a green preparation of suzetrigine [10]. The patent claims that the method has the advantages that the steps are shorter, the reaction is safer, the possible racemization risk caused by preparing acid into acyl chloride and the use and discharge of an ammonia methanol solution are avoided.

## 4. Mechanism of Action of Suzetrigine: Targeting Peripheral Nociception

**Figure 2.**
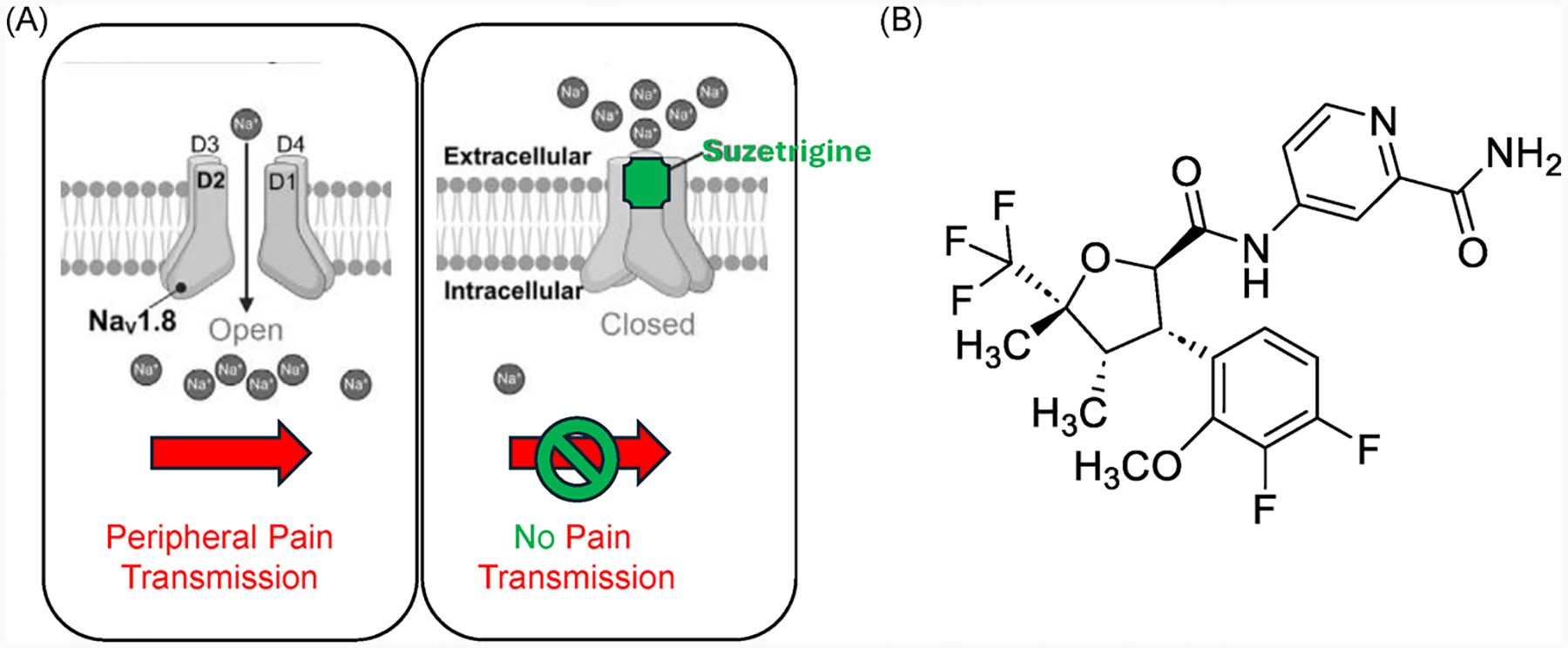
(**A**) The mechanism of action of suzetrigine (VX-548); (**B**) the chemical structure of suzetrigine.

Suzetrigine’s novel mechanism involves highly selective inhibition of the Nav1.8 sodium channel isoform, which is predominantly expressed in peripheral nociceptors (Figure 2) [11–15]. Unlike non-selective sodium channel blockers, suzetrigine binds specifically to the voltage-sensing domain 2 (VSD2) of the Nav1.8 sodium channel, stabilizing the channel in its closed state through allosteric modulation. This unique mechanism confers several therapeutic advantages: (1) peripheral restriction: minimal CNS penetration (brain-plasma ratio < 0.1) avoids opioid-like central effects; (2) exceptional selectivity: 31,000-fold greater affinity for Nav1.8 over other sodium channel subtypes (Nav1.1, Nav1.7, Nav1.9); and (3) functional selectivity: Inhibits pain signaling without causing complete neuronal blockade [11–15].

Recent studies have provided further insight into the mechanism of action of suzetrigine [16,17]. They show that suzetrigine displays a rare form of state dependence known as “reverse use-dependence,” in which its inhibitory effect can be reduced by repetitive or strong depolarizations—a phenomenon previously observed with other Nav1.8 inhibitors. Specifically, relief from inhibition during strong depolarizations occurs with a time constant of approximately 40 milliseconds and is not dependent on drug concentration. In contrast, reinhibition at resting (negative) voltages is closely tied to drug concentration, suggesting that relief involves drug dissociation from the channel, while reinhibition reflects rebinding. This pattern indicates that suzetrigine binds tightly to Nav1.8 channels in their resting state but has minimal affinity for channels with fully activated voltage sensors [16,17]. Recent structural studies using cryogenic electron microscopy (cryo-EM) and structure-based predictive modeling have also provided unprecedented insights into the structural pharmacology of Na_v_1.8. Nevertheless, the structure of Nav1.8 with VX-548 is yet to be determined [18].

## 5. Pharmacokinetics of Suzetrigine

Suzetrigine exhibits favorable pharmacokinetic properties for oral administration. After a single oral dose, the median time to maximum plasma concentration (Tmax) is approximately 3 h under fasting conditions, and this may be delayed to around 5 h when taken with food. Its effective half-life is about 23.6 h, allowing for twice-daily dosing to maintain therapeutic plasma levels [19,20]. The apparent volume of distribution is large, estimated at 495 L, indicating extensive distribution in peripheral tissues. Suzetrigine is highly protein-bound (99%), which contributes to a prolonged plasma half-life and reduced renal clearance. The apparent oral clearance is 13.9 L/h. Suzetrigine is primarily metabolized by cytochrome P450 3A (CYP3A) enzymes. Its major active metabolite, M6-SUZ, has approximately 3.7-fold lower potency against NaV1.8 compared to the parent compound. Both suzetrigine and M6-SUZ are excreted through feces (49.9%) and urine (44%), mostly in the form of metabolites [19,20].

Co-administration with a high- or moderate-fat meal delays the Tmax of both suzetrigine and M6-SUZ, but does not significantly affect the overall exposure (AUC or Cmax). Therefore, suzetrigine can be administered with or without food. No clinically significant differences in suzetrigine pharmacokinetics have been observed across age (18–75 years), sex, race, body weight (44–126 kg), or in patients with mild renal or hepatic impairment. However, in moderate hepatic impairment, exposure to suzetrigine increases by 1.3 to 1.5-fold, and caution is advised. The effects of severe hepatic or renal impairment (eGFR < 15 mL/min) are unknown. Strong CYP3A inhibitors (e.g., itraconazole) can increase suzetrigine exposure by 4.8-fold, while inducers like rifampin can reduce exposure by over 90%, necessitating contraindications or careful monitoring. Moderate CYP3A inhibitors (e.g., fluconazole) also increase exposure modestly. Suzetrigine itself may induce CYP3A, potentially affecting co-administered drugs [20,21]. No clinically significant differences in suzetrigine and M6-SUZ pharmacokinetics were observed when it was used concomitantly with proton pump inhibitor omeprazole. No clinically significant differences in the pharmacokinetics of oral digoxin (P-glycoprotein substrate), ethinyl estradiol (hormonal contraceptive), or levonorgestrel (hormonal contraceptive) were observed when used concomitantly with suzetrigine. Suzetrigine inhibits CYP2C8, CYP2C9, and CYP2C19, but is not expected to result in clinically significant drug interactions. Suzetrigine does not inhibit CYP1A2, CYP2B6, CYP2D6, and CYP3A enzymes, and M6-SUZ does not inhibit CYP1A2, CYP2B6, CYP2C8, CYP2C9, CYP2C19, CYP2D6, and CYP3A enzymes. Suzetrigine induces CYP3A and, to a lesser extent, CYP2B6, CYP2C8, CYP2C9, and CYP2C19. Suzetrigine does not induce CYP1A2. Furthermore, suzetrigine and its main metabolite are not substrates of BCRP, OATP1B1, or OATP1B3. Suzetrigine is not a P-gp substrate, but M6-SUZ is a P-gp substrate. Suzetrigine inhibits OATP1B1, OATP1B3, and OAT3 but is not expected to result in clinically significant drug interactions. Suzetrigine does not inhibit BCRP, OAT1, OCT2, MATE1, and MATE2/K transporters. M6-SUZ inhibits OATP1B1, OATP1B3, OAT1, and OAT3 but is not expected to result in clinically significant drug interactions. M6-SUZ does not inhibit P-glycoprotein, BCRP, OCT2, MATE1, and MATE2/K transporters.

## 6. Clinical Efficacy: Evidence from Pivotal Trials

The FDA approval of suzetrigine was based on results from two multicenter, randomized, double-blind, placebo- and active-controlled trials. Suzetrigine underwent two pivotal randomized controlled trials in participants undergoing abdominoplasty and bunionectomy (NCT04891132, NCT04891145). These are standard models in pain research due to the consistent pain trajectory post-surgery. Participants (aged 18 to 80 years with moderate or severe postoperative pain (≥4 on the Numeric Pain Rating Scale [NPRS]) received either high-dose suzetrigine (100 mg loading, 50 mg BID maintenance), low-dose suzetrigine, placebo, or hydrocodone bitartrate/acetaminophen (HB/APAP; 5/325 mg every 6 h). The primary endpoint for efficacy assessment was SPID48, which represents the time-weighted sum of the pain intensity difference over 48 h, with higher SPID48 values indicating better pain relief. Key secondary endpoints included comparing suzetrigine’s SPID48 with that of HB/APAP and assessing the time to at least a 2-point NPRS reduction from baseline [21–25].

In the abdominoplasty trial (*N* = 1118), suzetrigine achieved a significant reduction in the SPID48 score (Sum of Pain Intensity Difference over 48 h) compared to placebo: 48.4 (95% CI: 33.6, 63.1; *p* < 0.0001). There was also a 52% reduction in rescue medication use vs. placebo (*p* = 0.003). In the bunionectomy model (*N* = 1073), the difference was 29.3 (95% CI: 14.0, 44.6; *p* = 0.0002). Suzetrigine provided faster pain relief than placebo, with median times to a ≥2-point NPRS reduction of 119 min (abdominoplasty) and 240 min (bunionectomy) versus 480 min for placebo. Nevertheless, suzetrigine did not exhibit superior pain relief to HB/APAP, with SPID48 differences of 6.6 and −20.2 in the abdominoplasty and bunionectomy trials, respectively. Suzetrigine was generally well tolerated. Adverse event rates after abdominoplasty were 50% for suzetrigine, 56.3% for placebo, and 60.7% for HB/APAP. After bunionectomy, the rates were 31%, 35.2%, and 41.8%, respectively. Participants reported no serious adverse events related to suzetrigine. Together, these outcomes are considered both statistically and clinically significant, supporting the analgesic efficacy of suzetrigine in acute post-surgical pain [21–25].

To evaluate broader real-world applicability, a single-arm, 6-month, open-label safety extension study was conducted across the U.S. in 222 surgical and 34 non-surgical participants aged 18 to 80 with pain rated ≥4 on the NPRS or moderate/severe on the verbal categorical rating scale (VRS). Participants were experiencing moderate to severe acute pain from a broad range of surgical and non-surgical causes. Surgical cases were predominantly orthopedic (41.9%), plastic (37.4%), and otorhinolaryngologic (10.8%), while non-surgical cases included sprains and strains. Participants received suzetrigine (same dosing previously described) for up to 14 days or until their pain resolved. 83.2% of patients rated the drug as “good,” “very good,” or “excellent” in treating their pain. However, 73% of participants required supplemental NSAIDs, suggesting suzetrigine may be most effective as part of multimodal analgesia. Suzetrigine was generally safe; most adverse events were mild to moderate, most commonly headache, occurring in 7% of patients, and participants reported no serious adverse events related to the drug [21–25]. This study reinforced suzetrigine’s broad utility and tolerability outside of controlled experimental settings, although the lack of a comparator arm limits definitive conclusions about its comparative effectiveness.

It is worth emphasizing that some experts in the field appear to be skeptical of suzetrigine [26]. In this context, they argue that while suzetrigine is being hailed as a promising alternative for moderate-to-severe acute pain, its approval is based on limited and methodologically flawed evidence from two short-term surgical pain trials, raising questions about its real-world effectiveness and generalizability. Despite being marketed as safer and non-addictive, suzetrigine’s efficacy over placebo is clinically ambiguous, and concerns remain about biased industry-led studies, unreported NSAID use, and inadequate comparators. They also argue that the urgency to find opioid alternatives has led regulators to fast-track approvals, sometimes at the expense of scientific rigor, as seen in past cases like extended-release oxycodone and esketamine. While suzetrigine represents a new direction in pain management, its long-term role, especially in chronic pain, is uncertain. Accordingly, the critics argue that FDA must strike a careful balance between public health needs and scientific standards, ensuring robust post-market monitoring and thoughtful prioritization to avoid repeating past regulatory missteps.

## 7. Safety and Tolerability

Pooled safety data from 2191 participants across Phase 2–3 trials reveal a favorable profile. The most common (≥1%) side events were pruritus (2.1%), muscle spasms (1.3%), elevated CPK (1.1%), and rash (1.1%). Serious adverse events were postoperative hematoma (0.2%) and syncope (0.1%). Fortunately, no cases of respiratory depression or QTc prolongation (QT interval on an ECG) were reported. The drug has a low abuse liability. Likewise, abuse potential studies in humans were negative at 3× therapeutic dose. The medication has no reports of euphoria or withdrawal symptoms. As stated above, special considerations include potential CYP3A4 interactions as well as use in patients with hepatic impairment or renal impairment. The medication is contraindicated with strong CYP3A4 inhibitors (e.g., itraconazole). It requires a 50% dose reduction with moderate CYP3A4 inhibitors (e.g., fluconazole). Considering the Child–Pugh scoring system, which is used to assess the prognosis of chronic liver disease, mainly cirrhosis, the drug is to be avoided in Child–Pugh C (1–2 years survival of 45–35%; severe impairment), and its administration frequency is to be reduced in Child–Pugh B (1–2 years survival of 80–60%; moderate impairment). The medication is not recommended if eGFR < 15 mL/min [4,27–31].

Long-term animal studies to assess the carcinogenic potential of suzetrigine have not yet been conducted. However, suzetrigine was not found to be mutagenic in the bacterial reverse mutation assay (Ames test), nor was it clastogenic in either the in vitro micronucleus assay using human TK6 lymphoblastoid cells or the in vivo rat bone marrow micronucleus assay. In a female fertility study, female rats were administered suzetrigine orally at doses of 5, 10, and 15 mg/kg/day for at least 14 days prior to mating, continuing through mating and up to Gestation Day 7. These doses correspond to approximately 0.57, 1.6, and 2.2 times the steady-state maximum recommended human dose (MRHD) exposure based on AUC. At the highest dose (15 mg/kg/day), increased pre-implantation loss was observed. This effect is thought to be related to suzetrigine’s activity on the rat progesterone receptor, which is more sensitive to the drug than the human receptor, as demonstrated in in vitro studies. Importantly, no adverse effects on female fertility or early embryonic development were noted after a 4-week recovery period. The relevance of these findings to humans remains uncertain. In a separate male fertility study, male rats received oral doses of suzetrigine at 200, 600, and 1000 mg/kg/day for at least 28 days before and during mating. These exposures represent 3.6, 9.7, and 13.8 times the steady-state MRHD exposure based on AUC. No effects were observed on sperm parameters (motility, concentration, morphology), reproductive performance, or uterine outcomes (implantation count, viable implants, pre-implantation loss, early resorptions, or post-implantation loss) at any dose level [4,27–31].

## 8. Clinical Applications

Current evidence supports the use of suzetrigine for postoperative pain, particularly soft tissue (abdominoplasty) and orthopedic (bunionectomy) procedures. It is also used to alleviate acute musculoskeletal pain as an alternative to NSAIDs in high-risk patients. It is also promising in opioid-sparing regimens for patients with a substance use history. The dosing protocol can start with a 100 mg loading dose (fasting state), followed by 50 mg q12h (with/without food) as a maintenance dose. The drug is advised to be continuously used maximally for 14 days [4,31].

While promising, several questions remain regarding the use of suzetrigine. For example, no trials versus full-dose opioids to establish its comparative effectiveness exist [32]. There is an ongoing diabetic neuropathy trial (NCT04991178) for chronic pain use [33]. There are also concerns about its cost-effectiveness [34]. The future research should explore combination strategies with NSAIDs, gabapentinoids, or local anesthetics, which could enhance pain control. Post-marketing studies will refine its role in clinical practice regarding real-world outcomes in diverse populations. The next-generation Nav1.8 inhibitors may also offer improved efficacy [35,36].

## 9. Conclusions

The opioid crisis has underscored the urgent need for effective, non-addictive analgesics, yet innovation in pain management has lagged for decades. Suztrigine, the first FDA-approved selective Nav1.8 inhibitor, represents a notable pharmacological development in acute pain treatment. By targeting peripheral sodium channels with minimal CNS penetration, it avoids the sedation, euphoria, and respiratory depression associated with opioids while providing clinically meaningful pain relief. Pivotal Phase 3 trials demonstrated its superiority over placebo, with a significant improvement in pain scores (SPID48: +29.3 to +48.4, *p* < 0.0001) and an approximately 2 h onset of action. Its novel mechanism—allosteric inhibition of Nav1.8 without pore blockade—distinguishes it from traditional local anesthetics and offers a much-needed alternative in the opioid-sparing arsenal.

Despite its promise, suzetrigine is not without limitations. Its effect size, while statistically significant, remains modest compared to full-dose opioids, and most trial patients required rescue NSAIDs. Additionally, its pharmacokinetics pose challenges: strong CYP3A inhibitors (e.g., itraconazole) are contraindicated due to a nearly fivefold increase in drug exposure, and dose adjustments are needed with moderate inhibitors. The lack of long-term safety data restricts its use to 14-day courses, and its high cost (USD 15.50 per 50 mg tablet) may limit accessibility. However, its favorable safety profile—no abuse potential and only mild adverse effects like pruritus (2.1%) and muscle spasms (1.3%)—makes it a compelling option for opioid-avoidant patients, particularly in postoperative and acute musculoskeletal pain settings.

Looking ahead, suzetrigine’s role in pain management will likely expand as researchers explore its potential in chronic pain (e.g., diabetic neuropathy) and combination therapies with NSAIDs or local anesthetics. Its approval marks a turning point in analgesic development, proving that targeted non-opioid mechanisms can achieve meaningful pain relief. While it may not replace opioids in all scenarios, it offers a critical tool for reducing reliance on addictive medications. As post-marketing data accumulate, suzetrigine could redefine standards of care, balancing efficacy with safety in an era demanding alternatives to opioids. Clinicians should cautiously embrace its strengths—2 h onset of action, peripheral action, and low abuse risk—while remaining mindful of its drug interactions and cost constraints. Ultimately, suzetrigine is both an emerging option and a stepping stone toward a future where effective pain management no longer hinges on opioids.
